# Supplementary material for: A fault-intrusion-tolerant system and deadline-aware algorithm for scheduling scientific workflow in the cloud
Source: PeerJ Comput Sci. 2021 Nov 2;7:e747. doi: 10.7717/peerj-cs.747 (PMC8576554; doi:10.7717/peerj-cs.747)
Supplement: Supplemental Information 6 [file peerj-cs-07-747-s006.zip › Myproject1.1/docs/org/cloudbus/cloudsim/core/SimEntity.html]

SimEntity


JavaScript is disabled on your browser.


- Overview
- Package
- Class
- Use
- Tree
- Deprecated
- Index
- Help

- Prev Class
- Next Class

- Frames
- No Frames

- All Classes

- Summary:
- Nested |
- Field |
- Constr |
- Method

- Detail:
- Field |
- Constr |
- Method


org.cloudbus.cloudsim.core

## Class SimEntity

- java.lang.Object
- - org.cloudbus.cloudsim.core.SimEntity

- All Implemented Interfaces:
  :   java.lang.Cloneable

  Direct Known Subclasses:
  :   CloudInformationService, CloudSimExample8.GlobalBroker, CloudSimShutdown, ClusteringEngine, Datacenter, DatacenterBroker, NetDatacenterBroker, Switch, WorkflowEngine, WorkflowPlanner

  ---

    

  ```
  public abstract class SimEntity
  extends java.lang.Object
  implements java.lang.Cloneable
  ```

  This class represents a simulation entity. An entity handles events and can send events to other
  entities. When this class is extended, there are a few methods that need to be implemented:
  - `startEntity()` is invoked by the `Simulation` class when the simulation is
    started. This method should be responsible for starting the entity up.- `processEvent(SimEvent)` is invoked by the `Simulation` class whenever there is
      an event in the deferred queue, which needs to be processed by the entity.- `shutdownEntity()` is invoked by the `Simulation` before the simulation
        finishes. If you want to save data in log files this is the method in which the corresponding
        code would be placed.

  Since:
  :   CloudSim Toolkit 1.0

- - ### Field Summary

    Fields

    | Modifier and Type | Field and Description |
    | --- | --- |
    | `static int` | `FINISHED` The Constant FINISHED. |
    | `static int` | `HOLDING` The Constant HOLDING. |
    | `static int` | `RUNNABLE` The Constant RUNNABLE. |
    | `static int` | `WAITING` The Constant WAITING. |
  - ### Constructor Summary

    Constructors

    Constructor and Description

    `SimEntity(java.lang.String name)`

    Creates a new entity.
  - ### Method Summary

    Methods

    | Modifier and Type | Method and Description |
    | --- | --- |
    | `SimEvent` | `cancelEvent(Predicate p)` Cancel the first event matching a predicate waiting in the entity's future queue. |
    | `protected java.lang.Object` | `clone()` Get a clone of the entity. |
    | `protected SimEvent` | `getEventBuffer()` Gets the event buffer. |
    | `int` | `getId()` Get the unique id number assigned to this entity. |
    | `java.lang.String` | `getName()` Get the name of this entity. |
    | `SimEvent` | `getNextEvent()` Get the first event waiting in the entity's deferred queue, or if there are none, wait for an event to arrive. |
    | `SimEvent` | `getNextEvent(Predicate p)` Get the first event matching a predicate from the deferred queue, or if none match, wait for a matching event to arrive. |
    | `protected int` | `getState()` Gets the state. |
    | `int` | `numEventsWaiting()` Count how many events are waiting in the entity's deferred queue. |
    | `int` | `numEventsWaiting(Predicate p)` Count how many events matching a predicate are waiting in the entity's deferred queue. |
    | `void` | `pause(double delay)` Set the entity to be inactive for a time period. |
    | `abstract void` | `processEvent(SimEvent ev)` This method is invoked by the `Simulation` class whenever there is an event in the deferred queue, which needs to be processed by the entity. |
    | `void` | `run()` |
    | `void` | `schedule(int dest, double delay, int tag)` Send an event to another entity by id number and with **no** data. |
    | `void` | `schedule(int dest, double delay, int tag, java.lang.Object data)` Send an event to another entity by id number, with data. |
    | `void` | `schedule(java.lang.String dest, double delay, int tag)` Send an event to another entity through a port with a given name, with **no** data. |
    | `void` | `schedule(java.lang.String dest, double delay, int tag, java.lang.Object data)` Send an event to another entity through a port with a given name, with data. |
    | `void` | `scheduleFirst(int dest, double delay, int tag)` Send a high priority event to another entity by id number and with **no** data. |
    | `void` | `scheduleFirst(int dest, double delay, int tag, java.lang.Object data)` Send a high priority event to another entity by id number, with data. |
    | `void` | `scheduleFirst(java.lang.String dest, double delay, int tag)` Send a high priority event to another entity through a port with a given name, with **no** data. |
    | `void` | `scheduleFirst(java.lang.String dest, double delay, int tag, java.lang.Object data)` Send a high priority event to another entity through a port with a given name, with data. |
    | `void` | `scheduleFirstNow(int dest, int tag)` Send a high priority event to another entity by id number and with **no** data. |
    | `void` | `scheduleFirstNow(int dest, int tag, java.lang.Object data)` Send a high priority event to another entity by id number, with data. |
    | `void` | `scheduleFirstNow(java.lang.String dest, int tag)` Send a high priority event to another entity through a port with a given name, with **no** data. |
    | `void` | `scheduleFirstNow(java.lang.String dest, int tag, java.lang.Object data)` Send a high priority event to another entity through a port with a given name, with data. |
    | `void` | `scheduleNow(int dest, int tag)` Send an event to another entity by id number and with **no** data. |
    | `void` | `scheduleNow(int dest, int tag, java.lang.Object data)` Send an event to another entity by id number, with data. |
    | `void` | `scheduleNow(java.lang.String dest, int tag)` Send an event to another entity through a port with a given name, with **no** data. |
    | `void` | `scheduleNow(java.lang.String dest, int tag, java.lang.Object data)` Send an event to another entity through a port with a given name, with data. |
    | `SimEvent` | `selectEvent(Predicate p)` Extract the first event matching a predicate waiting in the entity's deferred queue. |
    | `protected void` | `send(int entityId, double delay, int cloudSimTag)` Sends an event/message to another entity by delaying the simulation time from the current time, with a tag representing the event type. |
    | `protected void` | `send(int entityId, double delay, int cloudSimTag, java.lang.Object data)` Sends an event/message to another entity by delaying the simulation time from the current time, with a tag representing the event type. |
    | `protected void` | `send(java.lang.String entityName, double delay, int cloudSimTag)` Sends an event/message to another entity by delaying the simulation time from the current time, with a tag representing the event type. |
    | `protected void` | `send(java.lang.String entityName, double delay, int cloudSimTag, java.lang.Object data)` Sends an event/message to another entity by delaying the simulation time from the current time, with a tag representing the event type. |
    | `protected void` | `sendNow(int entityId, int cloudSimTag)` Sends an event/message to another entity by delaying the simulation time from the current time, with a tag representing the event type. |
    | `protected void` | `sendNow(int entityId, int cloudSimTag, java.lang.Object data)` Sends an event/message to another entity by delaying the simulation time from the current time, with a tag representing the event type. |
    | `protected void` | `sendNow(java.lang.String entityName, int cloudSimTag)` Sends an event/message to another entity by delaying the simulation time from the current time, with a tag representing the event type. |
    | `protected void` | `sendNow(java.lang.String entityName, int cloudSimTag, java.lang.Object data)` Sends an event/message to another entity by delaying the simulation time from the current time, with a tag representing the event type. |
    | `protected void` | `setEventBuffer(SimEvent e)` Sets the event buffer. |
    | `protected void` | `setId(int id)` Sets the id. |
    | `protected void` | `setState(int state)` Sets the state. |
    | `abstract void` | `shutdownEntity()` This method is invoked by the `Simulation` before the simulation finishes. |
    | `abstract void` | `startEntity()` This method is invoked by the `Simulation` class when the simulation is started. |
    | `void` | `waitForEvent(Predicate p)` Wait for an event matching a specific predicate. |

    - ### Methods inherited from class java.lang.Object

      `equals, finalize, getClass, hashCode, notify, notifyAll, toString, wait, wait, wait`

- - ### Field Detail


    - #### RUNNABLE

      ```
      public static final int RUNNABLE
      ```

      The Constant RUNNABLE.

      See Also:
      :   Constant Field Values


    - #### WAITING

      ```
      public static final int WAITING
      ```

      The Constant WAITING.

      See Also:
      :   Constant Field Values


    - #### HOLDING

      ```
      public static final int HOLDING
      ```

      The Constant HOLDING.

      See Also:
      :   Constant Field Values


    - #### FINISHED

      ```
      public static final int FINISHED
      ```

      The Constant FINISHED.

      See Also:
      :   Constant Field Values
  - ### Constructor Detail


    - #### SimEntity

      ```
      public SimEntity(java.lang.String name)
      ```

      Creates a new entity.

      Parameters:
      :   `name` - the name to be associated with this entity
  - ### Method Detail


    - #### getName

      ```
      public java.lang.String getName()
      ```

      Get the name of this entity.

      Returns:
      :   The entity's name


    - #### getId

      ```
      public int getId()
      ```

      Get the unique id number assigned to this entity.

      Returns:
      :   The id number


    - #### schedule

      ```
      public void schedule(int dest,
                  double delay,
                  int tag,
                  java.lang.Object data)
      ```

      Send an event to another entity by id number, with data. Note that the tag `9999`
      is reserved.

      Parameters:
      :   `dest` - The unique id number of the destination entity
      :   `delay` - How long from the current simulation time the event should be sent
      :   `tag` - An user-defined number representing the type of event.
      :   `data` - The data to be sent with the event.


    - #### schedule

      ```
      public void schedule(int dest,
                  double delay,
                  int tag)
      ```

      Send an event to another entity by id number and with **no** data. Note that the tag
      `9999` is reserved.

      Parameters:
      :   `dest` - The unique id number of the destination entity
      :   `delay` - How long from the current simulation time the event should be sent
      :   `tag` - An user-defined number representing the type of event.


    - #### schedule

      ```
      public void schedule(java.lang.String dest,
                  double delay,
                  int tag,
                  java.lang.Object data)
      ```

      Send an event to another entity through a port with a given name, with data. Note that the
      tag `9999` is reserved.

      Parameters:
      :   `dest` - The name of the port to send the event through
      :   `delay` - How long from the current simulation time the event should be sent
      :   `tag` - An user-defined number representing the type of event.
      :   `data` - The data to be sent with the event.


    - #### schedule

      ```
      public void schedule(java.lang.String dest,
                  double delay,
                  int tag)
      ```

      Send an event to another entity through a port with a given name, with **no** data. Note
      that the tag `9999` is reserved.

      Parameters:
      :   `dest` - The name of the port to send the event through
      :   `delay` - How long from the current simulation time the event should be sent
      :   `tag` - An user-defined number representing the type of event.


    - #### scheduleNow

      ```
      public void scheduleNow(int dest,
                     int tag,
                     java.lang.Object data)
      ```

      Send an event to another entity by id number, with data. Note that the tag `9999`
      is reserved.

      Parameters:
      :   `dest` - The unique id number of the destination entity
      :   `tag` - An user-defined number representing the type of event.
      :   `data` - The data to be sent with the event.


    - #### scheduleNow

      ```
      public void scheduleNow(int dest,
                     int tag)
      ```

      Send an event to another entity by id number and with **no** data. Note that the tag
      `9999` is reserved.

      Parameters:
      :   `dest` - The unique id number of the destination entity
      :   `tag` - An user-defined number representing the type of event.


    - #### scheduleNow

      ```
      public void scheduleNow(java.lang.String dest,
                     int tag,
                     java.lang.Object data)
      ```

      Send an event to another entity through a port with a given name, with data. Note that the
      tag `9999` is reserved.

      Parameters:
      :   `dest` - The name of the port to send the event through
      :   `tag` - An user-defined number representing the type of event.
      :   `data` - The data to be sent with the event.


    - #### scheduleNow

      ```
      public void scheduleNow(java.lang.String dest,
                     int tag)
      ```

      Send an event to another entity through a port with a given name, with **no** data. Note
      that the tag `9999` is reserved.

      Parameters:
      :   `dest` - The name of the port to send the event through
      :   `tag` - An user-defined number representing the type of event.


    - #### scheduleFirst

      ```
      public void scheduleFirst(int dest,
                       double delay,
                       int tag,
                       java.lang.Object data)
      ```

      Send a high priority event to another entity by id number, with data. Note that the tag
      `9999` is reserved.

      Parameters:
      :   `dest` - The unique id number of the destination entity
      :   `delay` - How long from the current simulation time the event should be sent
      :   `tag` - An user-defined number representing the type of event.
      :   `data` - The data to be sent with the event.


    - #### scheduleFirst

      ```
      public void scheduleFirst(int dest,
                       double delay,
                       int tag)
      ```

      Send a high priority event to another entity by id number and with **no** data. Note that
      the tag `9999` is reserved.

      Parameters:
      :   `dest` - The unique id number of the destination entity
      :   `delay` - How long from the current simulation time the event should be sent
      :   `tag` - An user-defined number representing the type of event.


    - #### scheduleFirst

      ```
      public void scheduleFirst(java.lang.String dest,
                       double delay,
                       int tag,
                       java.lang.Object data)
      ```

      Send a high priority event to another entity through a port with a given name, with data.
      Note that the tag `9999` is reserved.

      Parameters:
      :   `dest` - The name of the port to send the event through
      :   `delay` - How long from the current simulation time the event should be sent
      :   `tag` - An user-defined number representing the type of event.
      :   `data` - The data to be sent with the event.


    - #### scheduleFirst

      ```
      public void scheduleFirst(java.lang.String dest,
                       double delay,
                       int tag)
      ```

      Send a high priority event to another entity through a port with a given name, with **no**
      data. Note that the tag `9999` is reserved.

      Parameters:
      :   `dest` - The name of the port to send the event through
      :   `delay` - How long from the current simulation time the event should be sent
      :   `tag` - An user-defined number representing the type of event.


    - #### scheduleFirstNow

      ```
      public void scheduleFirstNow(int dest,
                          int tag,
                          java.lang.Object data)
      ```

      Send a high priority event to another entity by id number, with data. Note that the tag
      `9999` is reserved.

      Parameters:
      :   `dest` - The unique id number of the destination entity
      :   `tag` - An user-defined number representing the type of event.
      :   `data` - The data to be sent with the event.


    - #### scheduleFirstNow

      ```
      public void scheduleFirstNow(int dest,
                          int tag)
      ```

      Send a high priority event to another entity by id number and with **no** data. Note that
      the tag `9999` is reserved.

      Parameters:
      :   `dest` - The unique id number of the destination entity
      :   `tag` - An user-defined number representing the type of event.


    - #### scheduleFirstNow

      ```
      public void scheduleFirstNow(java.lang.String dest,
                          int tag,
                          java.lang.Object data)
      ```

      Send a high priority event to another entity through a port with a given name, with data.
      Note that the tag `9999` is reserved.

      Parameters:
      :   `dest` - The name of the port to send the event through
      :   `tag` - An user-defined number representing the type of event.
      :   `data` - The data to be sent with the event.


    - #### scheduleFirstNow

      ```
      public void scheduleFirstNow(java.lang.String dest,
                          int tag)
      ```

      Send a high priority event to another entity through a port with a given name, with **no**
      data. Note that the tag `9999` is reserved.

      Parameters:
      :   `dest` - The name of the port to send the event through
      :   `tag` - An user-defined number representing the type of event.


    - #### pause

      ```
      public void pause(double delay)
      ```

      Set the entity to be inactive for a time period.

      Parameters:
      :   `delay` - the time period for which the entity will be inactive


    - #### numEventsWaiting

      ```
      public int numEventsWaiting(Predicate p)
      ```

      Count how many events matching a predicate are waiting in the entity's deferred queue.

      Parameters:
      :   `p` - The event selection predicate

      Returns:
      :   The count of matching events


    - #### numEventsWaiting

      ```
      public int numEventsWaiting()
      ```

      Count how many events are waiting in the entity's deferred queue.

      Returns:
      :   The count of events


    - #### selectEvent

      ```
      public SimEvent selectEvent(Predicate p)
      ```

      Extract the first event matching a predicate waiting in the entity's deferred queue.

      Parameters:
      :   `p` - The event selection predicate

      Returns:
      :   the simulation event


    - #### cancelEvent

      ```
      public SimEvent cancelEvent(Predicate p)
      ```

      Cancel the first event matching a predicate waiting in the entity's future queue.

      Parameters:
      :   `p` - The event selection predicate

      Returns:
      :   The number of events cancelled (0 or 1)


    - #### getNextEvent

      ```
      public SimEvent getNextEvent(Predicate p)
      ```

      Get the first event matching a predicate from the deferred queue, or if none match, wait for
      a matching event to arrive.

      Parameters:
      :   `p` - The predicate to match

      Returns:
      :   the simulation event


    - #### waitForEvent

      ```
      public void waitForEvent(Predicate p)
      ```

      Wait for an event matching a specific predicate. This method does not check the entity's
      deferred queue.

      Parameters:
      :   `p` - The predicate to match


    - #### getNextEvent

      ```
      public SimEvent getNextEvent()
      ```

      Get the first event waiting in the entity's deferred queue, or if there are none, wait for an
      event to arrive.

      Returns:
      :   the simulation event


    - #### startEntity

      ```
      public abstract void startEntity()
      ```

      This method is invoked by the `Simulation` class when the simulation is started. This
      method should be responsible for starting the entity up.


    - #### processEvent

      ```
      public abstract void processEvent(SimEvent ev)
      ```

      This method is invoked by the `Simulation` class whenever there is an event in the
      deferred queue, which needs to be processed by the entity.

      Parameters:
      :   `ev` - the event to be processed by the entity


    - #### shutdownEntity

      ```
      public abstract void shutdownEntity()
      ```

      This method is invoked by the `Simulation` before the simulation finishes. If you want
      to save data in log files this is the method in which the corresponding code would be placed.


    - #### run

      ```
      public void run()
      ```


    - #### clone

      ```
      protected final java.lang.Object clone()
                                      throws java.lang.CloneNotSupportedException
      ```

      Get a clone of the entity. This is used when independent replications have been specified as
      an output analysis method. Clones or backups of the entities are made in the beginning of the
      simulation in order to reset the entities for each subsequent replication. This method should
      not be called by the user.

      **Overrides:**
      :   `clone` in class `java.lang.Object`

      Returns:
      :   A clone of the entity

      Throws:
      :   `java.lang.CloneNotSupportedException` - the clone not supported exception


    - #### getState

      ```
      protected int getState()
      ```

      Gets the state.

      Returns:
      :   the state


    - #### getEventBuffer

      ```
      protected SimEvent getEventBuffer()
      ```

      Gets the event buffer.

      Returns:
      :   the event buffer


    - #### setState

      ```
      protected void setState(int state)
      ```

      Sets the state.

      Parameters:
      :   `state` - the new state


    - #### setId

      ```
      protected void setId(int id)
      ```

      Sets the id.

      Parameters:
      :   `id` - the new id


    - #### setEventBuffer

      ```
      protected void setEventBuffer(SimEvent e)
      ```

      Sets the event buffer.

      Parameters:
      :   `e` - the new event buffer


    - #### send

      ```
      protected void send(int entityId,
              double delay,
              int cloudSimTag,
              java.lang.Object data)
      ```

      Sends an event/message to another entity by delaying the simulation time from the
      current time, with a tag representing the event type.

      Parameters:
      :   `entityId` - the id number of the destination entity
      :   `delay` - how long from the current simulation time the event should be sent. If delay is
          a negative number, then it will be changed to 0
      :   `cloudSimTag` - an user-defined number representing the type of an event/message
      :   `data` - A reference to data to be sent with the event


    - #### send

      ```
      protected void send(int entityId,
              double delay,
              int cloudSimTag)
      ```

      Sends an event/message to another entity by delaying the simulation time from the
      current time, with a tag representing the event type.

      Parameters:
      :   `entityId` - the id number of the destination entity
      :   `delay` - how long from the current simulation time the event should be sent. If delay is
          a negative number, then it will be changed to 0
      :   `cloudSimTag` - an user-defined number representing the type of an event/message


    - #### send

      ```
      protected void send(java.lang.String entityName,
              double delay,
              int cloudSimTag,
              java.lang.Object data)
      ```

      Sends an event/message to another entity by delaying the simulation time from the
      current time, with a tag representing the event type.

      Parameters:
      :   `entityName` - the name of the destination entity
      :   `delay` - how long from the current simulation time the event should be sent. If delay is
          a negative number, then it will be changed to 0
      :   `cloudSimTag` - an user-defined number representing the type of an event/message
      :   `data` - A reference to data to be sent with the event


    - #### send

      ```
      protected void send(java.lang.String entityName,
              double delay,
              int cloudSimTag)
      ```

      Sends an event/message to another entity by delaying the simulation time from the
      current time, with a tag representing the event type.

      Parameters:
      :   `entityName` - the name of the destination entity
      :   `delay` - how long from the current simulation time the event should be sent. If delay is
          a negative number, then it will be changed to 0
      :   `cloudSimTag` - an user-defined number representing the type of an event/message


    - #### sendNow

      ```
      protected void sendNow(int entityId,
                 int cloudSimTag,
                 java.lang.Object data)
      ```

      Sends an event/message to another entity by delaying the simulation time from the
      current time, with a tag representing the event type.

      Parameters:
      :   `entityId` - the id number of the destination entity
      :   `delay` - how long from the current simulation time the event should be sent. If delay is
          a negative number, then it will be changed to 0
      :   `cloudSimTag` - an user-defined number representing the type of an event/message
      :   `data` - A reference to data to be sent with the event


    - #### sendNow

      ```
      protected void sendNow(int entityId,
                 int cloudSimTag)
      ```

      Sends an event/message to another entity by delaying the simulation time from the
      current time, with a tag representing the event type.

      Parameters:
      :   `entityId` - the id number of the destination entity
      :   `delay` - how long from the current simulation time the event should be sent. If delay is
          a negative number, then it will be changed to 0
      :   `cloudSimTag` - an user-defined number representing the type of an event/message


    - #### sendNow

      ```
      protected void sendNow(java.lang.String entityName,
                 int cloudSimTag,
                 java.lang.Object data)
      ```

      Sends an event/message to another entity by delaying the simulation time from the
      current time, with a tag representing the event type.

      Parameters:
      :   `entityName` - the name of the destination entity
      :   `delay` - how long from the current simulation time the event should be sent. If delay is
          a negative number, then it will be changed to 0
      :   `cloudSimTag` - an user-defined number representing the type of an event/message
      :   `data` - A reference to data to be sent with the event


    - #### sendNow

      ```
      protected void sendNow(java.lang.String entityName,
                 int cloudSimTag)
      ```

      Sends an event/message to another entity by delaying the simulation time from the
      current time, with a tag representing the event type.

      Parameters:
      :   `entityName` - the name of the destination entity
      :   `delay` - how long from the current simulation time the event should be sent. If delay is
          a negative number, then it will be changed to 0
      :   `cloudSimTag` - an user-defined number representing the type of an event/message


- Overview
- Package
- Class
- Use
- Tree
- Deprecated
- Index
- Help

- Prev Class
- Next Class

- Frames
- No Frames

- All Classes

- Summary:
- Nested |
- Field |
- Constr |
- Method

- Detail:
- Field |
- Constr |
- Method
